# Association of sleep behavior with depression: a cross-sectional study in northwestern China

**DOI:** 10.3389/fpsyt.2023.1171310

**Published:** 2023-06-23

**Authors:** Jingchun Liu, Suixia Cao, Yating Huo, Huimeng Liu, Yutong Wang, Binyan Zhang, Kun Xu, Peiying Yang, Lingxia Zeng, Shaonong Dang, Hong Yan, Baibing Mi

**Affiliations:** ^1^Department of Epidemiology and Biostatistics, School of Public Health, Xi’an Jiaotong University Health Science Center, Xi’an, Shaanxi, China; ^2^Global Health Institute, School of Public Health, Xi’an Jiaotong University Health Science Center, Xi’an, Shaanxi, China; ^3^Key Laboratory for Disease Prevention and Control and Health Promotion of Shaanxi Province, School of Public Health, Xi’an Jiaotong University Health Science Center, Xi’an, Shaanxi, China; ^4^Shaanxi Open Sharing Platform of Critical Disease Prevention and Big Health Data Science, School of Public Health, Xi’an Jiaotong University Health Science Center, Xi’an, Shaanxi, China; ^5^Department of Occupational and Environmental Health, School of Public Health, Xi’an Jiaotong University Health Science Center, Xi’an, Shaanxi, China

**Keywords:** depression, sleep duration, sleep problem, dose-response relation, population-based study, short sleep

## Abstract

**Background:**

This study aimed to examine the association between sleep duration, sleep problems, and depression in Northwest China.

**Method:**

Depression was diagnosed at the hospital and self-reported by the participants in the baseline survey. Sleep duration and problems, including difficulty initiating and maintaining sleep, early morning awakening, daytime dysfunction, use of sleeping pills or drugs, and any sleep problems, were obtained by a self-reported questionnaire. Logistic regression was used to estimate odds ratios (ORs) with corresponding 95% confidence intervals (CIs) for exploring the association between sleep duration, sleep problems, and depression, adjusting for demographic and socioeconomic characteristics and health behaviors. The association between depression and sleep duration was also evaluated continuously with restricted cubic spline curves based on logistic models.

**Results:**

36,515 adults from Regional Ethnic Cohort Study in Northwest China were included. About 24.04% of participants reported short sleep duration (<7 h), and 15.64% reported long sleep duration (≥9 h). Compared with standard sleep duration (7–9 h), short sleep duration was associated with a higher risk of depression (OR: 1.69, 95%CI: 1.26–2.27, *p* = 0.001). Self-reported sleep problems were also related to four times depression risk increased (OR: 4.02, 95%CI: 3.03–5.35, *p* < 0.001) compared with no sleep problems. In addition, a nonlinear relationship was found between sleep duration and depression after adjusting covariates (*p* = 0.043).

**Conclusion:**

Sleep duration and sleep problems are associated with depression. Enough sleep time and healthy sleep habits in life course might be a practical health promotion approach to reduce depression risk in Northwest Chinese adults. A further study from cohort study is needed to verify the temporal association.

## 1. Introduction

Depression devastates effects on people’s mental health and well-being, lowering their quality of life and increasing their risk of disability and premature death (1–3). The 12-month prevalence of depression is roughly 6% worldwide, and the lifetime prevalence is over 10% (4). A total of 258 million new instances of depression have been reported globally in the previous 30 years, as reported by the Global Burden of Disease research (5). Currently, major depression is the third most significant cause of disability worldwide, but the WHO predicts that by the year 2030, it will be the leading cause of disability worldwide. The all-age prevalence rate and the disability-adjusted life-years (DALYs) rate for depression is increasing in China, making it a severe public health problem (6).

Previous research has established strong associations between sociodemographic and behavioral factors and depression (7, 8). Managing destructive health behaviors, for instance, sleep duration may reduce the risk of depression (9). Recent findings from a meta-analysis confirmed that both short and long sleep duration was strongly related to the increased risk of depression. However, the majority of studies were conducted in the United States, Japan, and Russia (10–12). This may limit the generalizability of the findings to other countries. Besides, some studies explored the association in China, but the results needed to be more consistent (13, 14).

Notably, the 12-month and lifetime prevalence of depression is higher in central and western China (15). In fact, due to the diagnostic method, the prevalence and burden of depression in China may be higher than anticipated (16). Furthermore, it has been reported that Chinese residents sleep an average of 7.06 h per day, with variations across different regions (17). This suggests that there may be cultural factors that influence sleep patterns. Previous studies exploring the association between sleep duration and depression have mainly been conducted in central and eastern China (18, 19). There is limited evidence regarding the relationship between depression and sleep habits in western China.

To address these knowledge gaps in this study, we aim to explore the relationship between sleep duration, sleep problems, and depression using the Shaanxi baseline dataset of the Regional Ethnic Cohort Study (RECS) in Northwest China.

## 2. Methods

### 2.1. Participants and procedures

This cross-sectional data analysis was based on the baseline enrollment survey of the RECS in Shaanxi Province. RECS was a community, population-based, prospective observational study, with detailed information on the design and methodology, described elsewhere (20). A questionnaire survey, physical examination, biological sample collection, and laboratory testing were conducted in the study. The Human Research Ethics Committee of the Xi’an Jiaotong University Health Science Center approved this study (No: XJTU2016-411), and all the participants provided written informed consent.

From the original sample of 48,025 participants, we excluded 10,168 participants who had missing sleep duration or covariate information. After excluding an additional 1,342 participants from the analysis, 36,515 participants were included in the final analysis (Supplementary Figure 1). The excluded participants comprised those who reported sleep duration and daily work time of more than 24 h (*N* = 190), sleep duration of more than 24 h (*N* = 35), and those with implausible outlying data (e.g., BMI < 13 kg/m^2^ or physical activity [PA] = 0 MET-h/d; *N* = 1,117).

### 2.2. Sleep duration and sleep problems

Sleep duration was measured by self-report and included both nighttime sleep duration and naps. Participants were asked, “On average, how many hours do you sleep per night?” Respondents could report sleep duration in 1-h increments. Participants who reported taking naps were also asked, “On average, how many hours do you nap?” The possible responses to this question were within half an hour, half an hour to an hour, 1 to 2 h, and over 2 h, and we assigned 0.5, 0.75, 1.5, and 2.5 h, respectively. The total sleep duration was categorized as less than 7, 7 to 9, and 9 or more hours, with 7 to 9 h of sleep as the reference group. The cut-off points were based on previous studies (21).

Sleep problems were measured by self-report, including difficulty initiating and maintaining sleep, early morning awakening, use of sleeping pills or drugs, and daytime dysfunction. Participants were asked whether they had experienced these four sleep problems in the past month and were then classified into the corresponding group. For example, people who reported “having trouble falling asleep (sleep onset latency ≥30 min) after going to bed or waking up in the middle of the night at least 3 days a week” were classified as having difficulty initiating and maintaining sleep. The definition of sleep problems details was presented in Supplementary Table 1. Participants who reported one or more of the four sleep problems mentioned above were classified as having any sleep problems.

### 2.3. Depression

Depression was the primary outcome diagnosed at a hospital and self-reported by participants in the baseline survey. The outcome was obtained from the following self-reported question: “Have you ever been diagnosed with depression by a doctor of hospitals that is above district level? (yes/no).” The outcome was dichotomized into “depression participant” or “non-depression participant.”

### 2.4. Covariates

Demographic and socioeconomic characteristics included age (continuous, years), gender (male vs. female), level of education (primary school and below, middle school, or university and higher), occupation (yes vs. no), and household income (<10,000, 10,000–49,999, 50,000–99,999, and ≥100,000 yuan [CNY]/year). Trained staff measured body weight and height using calibrated instruments at baseline, and body mass index (BMI) was calculated as weight in kilograms divided by the height in meters squared. Based on the recommended cut-off points for Chinese adults (22), we categorized BMI into four groups: underweight (BMI < 18.5 kg/m^2^), normal weight (18.5 kg/m^2^ ≤ BMI < 24 kg/m^2^), overweight (24 kg/m^2^ ≤ BMI < 28 kg/m^2^), and obesity (BMI ≥ 28 kg/m^2^). Health behaviors included alcohol consumption (non-drinker vs. drinker), smoking status (non-smoker vs. smoker), tea consumption (non-tea drinker vs. tea drinker), coffee consumption (non-coffee drinker vs. coffee drinker), physical activity estimated as the metabolic equivalent task (continuous, Metabolic equivalent hours per day [MET-h/d]) (23). In addition, the night shift (yes vs. no) is also included in the model as an important covariate.

### 2.5. Statistic analysis

Characteristics of participants were described according to whether they had depression, using mean ± standard deviation (SD) and frequency (percentage) for continuous and categorical variables, respectively. A t-test or chi-square test was used to test differences in these variables.

Consider that depression event was rare in the study population, and penalized maximum likelihood logistic regression models were used to estimate the association between sleep behavior (including sleep duration and problems) and depression (24, 25). We constructed three models. Model 1 was a crude model without adjusting any other covariates. In addition, model 2 was adjusted for demographic and socioeconomic characteristics. Finally, model 3 further included health behaviors and the night shift. It is worth pointing out that the association between depression and sleep duration was also evaluated on a continuous scale with restricted cubic spline (RCS) curves based on logistic models. Besides, to explore whether participants who report sleep problems have an additional risk, we divided the three sleep duration groups into whether they had sleep problems, such as short sleep duration with or without any sleep problems, and then explored the relationship between sleep problems and depression.

We conducted several sensitivity and subgroup analyses to examine the robustness of our findings. In sensitivity analyses, we restricted participants who reported sleep duration between 4 and 12 h (*n* = 35,838) and performed multiple imputations of missing covariates before analysis (*n* = 42,506). In subgroup analyses, we further examined the association of sleep duration and subtypes of sleep problems with depression in age groups (≤60 and >60 years) (26, 27) and region groups (urban and rural regions). Participants were also grouped by season (spring, summer, autumn, and winter) at the time of the interview.

All statistical analyses were performed using the software of SAS version 9.4 (SAS Institute, Inc., Cary, NC), and all *p*-values refer to two-tailed tests. Statistical significance was set at *p* < 0.05.

## 3. Results

There were 36,515 participants in our analyses, including 62.01% females with a mean age of 51.10 ± 12.70 years. Baseline characteristics were summarized by depression status (Table 1). Compared with participants who did not report a diagnosis of depression, individuals with depression were more likely to have abnormal BMI, consume coffee, tea, and alcohol, exercise less, be more educated, are employed in nightshift work, and have abnormal sleep duration or sleep problems.

**Table 1:** Baseline characteristics of the study population according to depression.

Characteristics	Non-depression	Depression	*p* value
No. of participants	36,306	209	
Mean ± SD			
Age, years	51.16 ± 0.13	44.95 ± 1.78	<0.001
Physical activity (MET-h/d)	18.45 ± 0.13	19.97 ± 1.49	0.046
*n* (%)			
Sex			
Male	13,800 (38.01)	71 (33.97)	0.230
Female	22,506 (61.99)	138 (66.03)	
BMI			
Underweight	1,235 (3.40)	12 (5.74)	0.046
Normal	18,427 (50.75)	118 (56.46)	
Overweight	12,971 (35.73)	59 (28.23)	
Obesity	3,673 (10.12)	20 (9.57)	
Education level			
Primary and below	11,942 (32.89)	26 (12.44)	<0.001
Middle	14,392 (39.64)	49 (23.44)	
College	9,972 (27.47)	134 (64.11)	
Occupation			
No	8,840 (24.35)	27 (12.92)	0.004
Yes	27,466 (75.65)	182 (87.08)	
Household income (Yuan/year)			
<10,000	4,128 (11.37)	24 (11.48)	<0.001
10,000–49,999	18,278 (50.34)	53 (25.36)	
50,000–100,000	6,702 (18.46)	44 (21.05)	
>100,000	7,198 (19.83)	88 (42.11)	
Coffee			
No	29,914 (82.39)	125 (59.81)	<0.001
Yes	6,392 (17.61)	84 (40.19)	
Drinking			
No	25,112 (69.17)	111 (53.11)	<0.001
Yes	11,194 (30.83)	98 (46.89)	
Smoking			
No	29,103 (80.16)	166 (79.43)	0.791
Yes	7,203 (19.84)	43 (20.57)	
Tea			
No	20,471 (56.38)	79 (37.8)	<0.001
Yes	15,835 (43.62)	130 (62.2)	
Nightshift			
No	33,518 (92.32)	181 (86.60)	<0.001
Yes	2,788 (7.68)	28 (13.40)	
Any sleep problems^a^			
No	26,299 (72.44)	73 (34.93)	<0.001
Yes	10,007 (27.56)	136 (65.07)	
Difficulty initiating and maintaining sleep			
No	28,706 (79.07)	108 (51.67)	<0.001
Yes	7,600 (20.93)	101 (48.33)	
Early morning awaking			
No	31,395 (86.47)	143 (68.42)	<0.001
Yes	4,911 (13.53)	66 (31.58)	
Use of sleeping pills or drugs			
No	35,452 (97.65)	179 (85.65)	<0.001
Yes	854 (2.35)	30 (14.35)	
Daytime dysfunction			
No	33,757 (92.98)	152 (72.73)	<0.001
Yes	2,549 (7.02)	57 (27.27)	
Sleep duration (h)			
<7	8,727 (24.04)	70 (33.49)	0.001
7 ~ 9	21,902 (60.33)	122 (58.37)	
≥9	5,677 (15.64)	17 (8.13)	

SD, standard deviation; BMI, body mass index; MET-h/d, Metabolic equivalent of energy.

a Include difficulty initiating and maintaining sleep, early morning waking, use of sleeping pills or drugs and daytime dysfunction.

**Figure 1:**
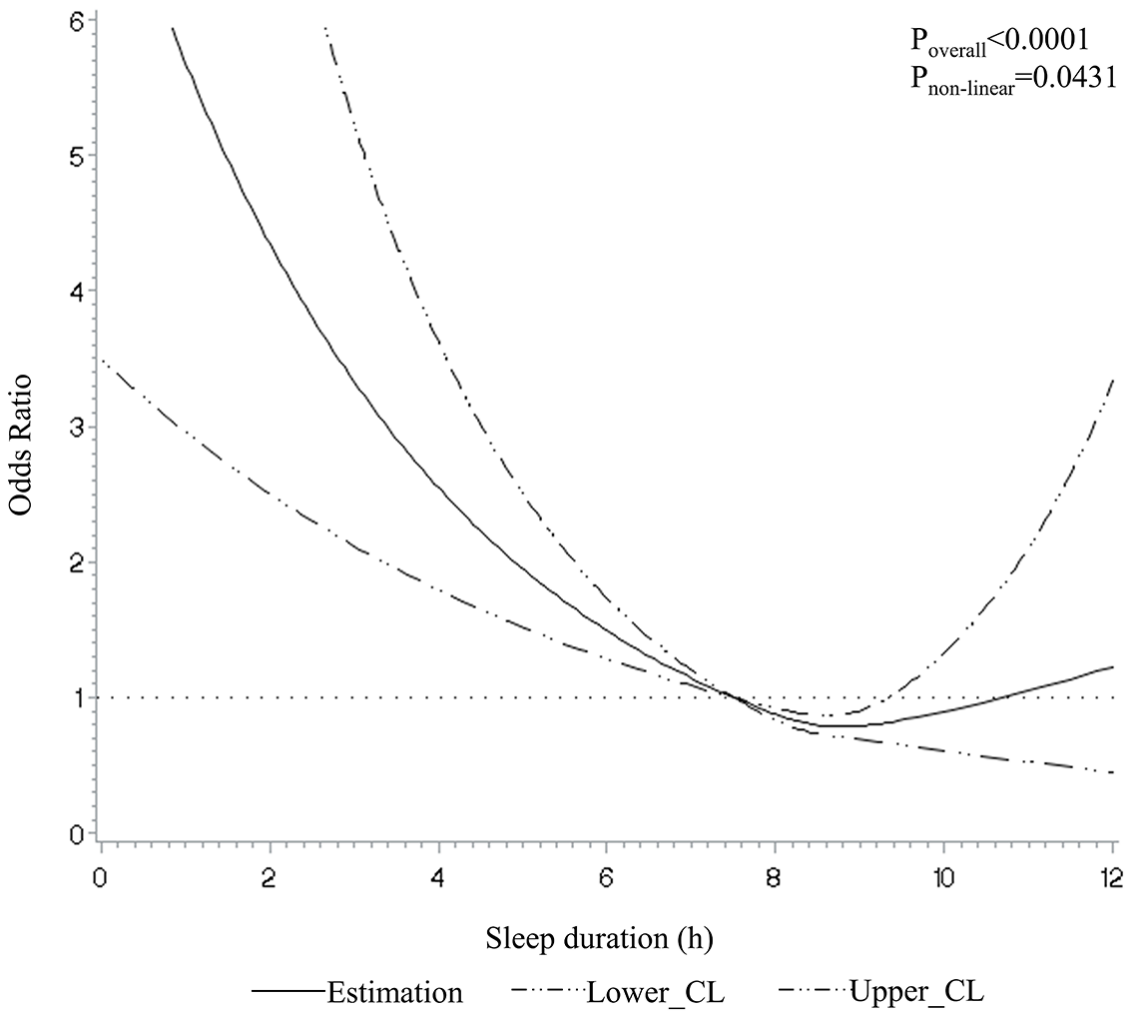
Dose–response relationship between sleep duration and depression in restricted cubic spline among all participants. CL, confidence limit. A 7.5-h sleep duration was used as the reference. The model was adjusted for sex, age, BMI, career, household income, educational level, drinking, smoking, tea, coffee, nightshift, and physical activity.

The associations between sleep duration and sleep problems with depression are illustrated in Table 2. Without adjustment for any covariates, participants with daily sleep durations shorter than 7 h had a 44% higher risk of depression (OR = 1.44, 95% CI: 1.08–1.94) compared to those without depression, while longer sleep durations (≥9 h) were associated with a lower risk of depression (OR = 0.55, 95% CI: 0.33–0.91). After adjustment for demographic and socioeconomic characteristics, the negative relationship of shorter daily sleep and depression persisted, but the protective effect of longer was not observed. In addition, the fully adjusted model revealed that short sleep duration was associated with higher odds of depression (OR = 1.69, 95% CI: 1.26–2.27). Nonlinear associations between sleep duration and depression were also explored in Figure 1, where short sleep duration was associated with higher depression risks (*p* = 0.043).

**Table 2:** Odds ratios (95% CI) of depression across sleep duration and sleep problem for all participants.

	Model 1		Model 2		Model 3	
	OR (95%CI)	*p* value	OR (95%CI)	*p* value	OR (95%CI)	*p* value
Sleep duration (h)						
7 ~ 9	Reference		Reference		Reference	
<7	1.44 (1.08,1.94)	0.014	1.71 (1.27,2.30)	<0.001	1.69 (1.26,2.27)	0.001
≥9	0.55 (0.33,0.91)	0.020	0.81 (0.49,1.34)	0.413	0.80 (0.49,1.32)	0.392
Difficulty initiating and maintaining sleep						
No	Reference		Reference		Reference	
Yes	3.53 (2.69,4.64)	<0.001	3.15 (2.40,4.14)	<0.001	3.13 (2.39,4.10)	<0.001
Early morning awaking						
No	Reference		Reference		Reference	
Yes	2.96 (2.21,3.97)	<0.001	2.75 (2.06,3.69)	<0.001	2.76 (2.07,3.70)	<0.001
Use of sleeping pills or drugs						
No	Reference		Reference		Reference	
Yes	7.05 (4.77,10.41)	<0.001	8.17 (5.46,12.22)	<0.001	8.21 (5.49,12.27)	<0.001
Daytime dysfunction						
No	Reference		Reference		Reference	
Yes	4.99 (3.68,6.78)	<0.001	3.76 (2.75,5.12)	<0.001	3.71 (2.72,5.06)	<0.001
Any sleep problems						
No	Reference		Reference		Reference	
Yes	4.88 (3.67,6.49)	<0.001	4.03 (3.03,5.36)	<0.001	4.02 (3.03,5.35)	<0.001

OR, odds ratio; CI, confidence interval. Model 1: crude model. Model 2: adjusted for sex, age, BMI, career, household income, and educational level. Model 3: additionally adjusted drinking, smoking, tea, coffee, nightshift, and physical activity.

Sleep problems were associated with increased odds of depression (OR = 4.02, 95% CI: 3.03–5.35), independent of covariates. We examined the effects of four types of sleep problems on depression in a fully adjusted model. In comparison with those reported sleep problems less than three times per week in the last month, participants who experienced sleep problems 3 or more times a week were associated with a greater likelihood of having depression [difficulty initiating and maintaining sleep (OR = 3.13, 95% CI: 2.39–4.10), early morning waking (OR = 2.76, 95% CI: 2.07–3.70), use of sleeping pills or drugs (OR = 8.21, 95% CI: 5.49–12.27), daytime dysfunction (OR = 3.71, 95% CI: 2.72–5.06)]. As shown in Table 3, considering the combined effect of sleep duration and sleep problems, participants with sleep problems in all three sleep duration groups had a higher risk of depression compared to those without any sleep problems (OR = 3.43, 95% CI: 2.40–4.90 for 7 ~ 9 h, OR = 4.45, 95% CI: 2.48–7.98 for <7 h, and OR = 5.29, 95% CI: 2.26–12.38 for ≥9 h).

**Table 3:** The combination effect of sleep duration and sleep problems for depression.

Combination of sleep duration and sleep problems	Model 1		Model 2		Model 3	
	OR (95%CI)	*p* value	OR (95%CI)	*p* value	OR (95%CI)	*p* value
7 ~ 9 (h)						
Without sleep problems	Reference		Reference		Reference	
With sleep problems	4.62 (3.23,6.60)	<0.001	3.49 (2.44,4.99)	<0.001	3.43 (2.40,4.90)	<0.001
<7 (h)						
Without sleep problems	Reference		Reference		Reference	
With sleep problems	4.26 (2.31,7.86)	<0.001	4.32 (2.38,7.81)	<0.001	4.45 (2.48,7.98)	<0.001
≥9 (h)						
Without sleep problems	Reference		Reference		Reference	
With sleep problems	9.12 (3.56,23.36)	<0.001	5.64 (2.35,13.56)	0.001	5.29 (2.26,12.38)	0.001

OR, odds ratio; CI, confidence interval. Model 1: crude model. Model 2: adjusted for sex, age, BMI, career, household income, and educational level. Model 3: additionally adjusted drinking, smoking, tea, coffee, nightshift, and physical activity.

Age group analysis revealed that the association between short sleep duration and depression was evident among participants younger than 60 years of age, but not in participants aged over 60 years old. The odds ratio (OR) for depression was 2.11 (95% CI: 1.20–3.73) for participants aged 60 years or younger, and 2.01 (95% CI: 0.68–5.91) for participants aged over 60 years. We observed similar results for the association between short sleep duration and depression in participants who lived in rural or urban areas (OR_rural_ = 4.54, 95% CI: 1.35–15.19; OR_urban_ = 1.81, 95% CI: 1.03–3.18). In the season group analysis, participants interviewed in the summer had a higher risk of depression (OR = 2.64, 95% CI: 1.24–5.63). The association between sleep problems and depression did not vary by age group and region. However, we did not observe an association between difficulty initiating and maintaining sleep and depression in participants interviewed in the winter (Supplementary Tables 2–4).

Sensitivity analysis revealed that the association between sleep behavior and depression did not change when restricting the analysis to participants with sleep duration between 4 to 12 h (Supplementary Table 5). However, performing multiple imputations of missing covariates before analysis showed that long sleep duration was likely to reduce the risk of depression (Supplementary Table 6). Maximum likelihood logistic regression models also showed consistent results (Supplementary Tables 7–13).

## 4. Discussion

In this study, 24.04% of the 36,515 participants had short sleep duration and 27.78% reported any sleep problems. This is consistent with the findings of other studies, which have shown that short sleep duration and sleep problems are common in China. For example, a nationally representative study found that 23.09% of middle-aged and elderly people slept less than 7 h, and 16.75% had any sleep problems (18). A study from central and eastern China found that 17.79% of the subjects had insufficient sleep (<7 h) (28). However, a study from southwest China found that over 30% of participants were categorized in the short sleep group. This study only calculated night sleep time rather than total sleep time (29). The China Sleep Report 2022 found that the Northwest region has the lowest sleep quality index, which was calculated by Pittsburgh Sleep Quality Index (PSQI) (17). This suggests that participants in northwest China might have shorter sleep duration and more sleep problems. This might be due to a variety of factors, including variation in diet habit, economy level, urbanization level, rhythm of life, and day length (13, 30, 31).

This present study found a nonlinear association between sleep duration and depression, and we only found a significant association between short sleep duration and depression. Although meta-analysis and a recent study suggested that long sleep duration also increases the risk of depression (32, 33), our study did not support this finding. However, results of this study are consistent with the findings of other longitudinal studies, which have shown that short sleep duration is a risk factor for depression in people over the age of 45 (34, 35). Furthermore, Northern Manhattan Study also suggested there was no cross-sectional or prospective association between long sleep duration (≥9 h) and depressive symptoms when compared to the reference (<6 h) (36). The plausible reason for this difference between long sleep duration and depression risk may be caused by the studied population, for instance, age and gender. In a cross-sectional analysis stratified by age group and gender, the association between long sleep duration and depressive symptoms was only found in women and people aged 55–65 (28). This discrepancy may contribute to the fewer participants with long sleep duration in our study.

The study’s finding of the significant association between sleep problems and the risk of depression is consistent with existing evidence (37), in which the researcher found that daytime sleepiness and night waking at 15 years old were associated with the risk of depressive symptoms at age 24 (daytime sleepiness: OR = 1.38, 95% CI: 1.16–1.64; night waking: OR = 1.13, 95% CI: 1.02–1.26). Besides, a study was conducted among depressed older people in the Netherlands. This study suggested that sleep disturbances, including trouble falling asleep, waking up several times a night, waking up earlier than planned, and trouble getting back to sleep after waking up too early, are highly prevalent in patients with late-life depression and independently correlated with the severity of depression (38). The finding in our study was also in line with the meta-analysis (39). It is worth discussing that previous studies do not consider using sleeping pills as a sleep problem (18). However, according to the effect of cardiovascular disease (40), we think these participants who use medication to help sleep suffer from more severe sleep problems.

We found that individuals who reported sleep problems were more likely to associate with a higher risk. Research showed that long sleep experienced with severe sleep problems at baseline was not linked to future depressive symptoms (34). The difference might be explained by the different methods used to define the severity of sleep problems and the different reference groups. In our study, sleep problems might be more severe. Members of the reference group did not have any sleep problems. Other studies have shown that depression is more common in people with sleep deprivation and insomnia (41). Therefore, it is necessary to study further the combined effect of sleep duration and sleep problems.

We found an age-specific association between short sleep duration and depression in participants younger than 60 years of age, but not in participant aged 60 years or older. Previous research on elder also found no significant association between sleep restriction and depression (34). One potential explanation for this finding is that the current study, had a small number of participants who slept for more than 9 h per night, which may have limited the power to detect an association with depression (36). Another reason for this discrepancy of findings linking long sleep hours with depression risk may be differences in sleep measurement (objective vs. subjective). We did not find an association between short sleep duration and depression in participants interviewed in winter, which is inconsistent with previous findings that the highest proportion of major depressive episodes (MDEs) occur in winter and the lowest proportions occur in summer (42). This may be because over 80% of participants in the current study were interviewed in summer and autumn.

However, our study had several limitations. Firstly, the data of this present study comes from the baseline survey of the RECS. Because of the cross-sectional study design, causal relationships could not be inferred due to the cross-sectional design. Secondly, the prevalence of depression in our study is lower than that reported in the previous study (15). The most likely reason is that participants voluntarily enrolled in the present study may have better mental conditions than the general population. Another plausible explanation is that depression is self-reported, which means that the participant is answering the question based on their own knowledge and experience. It is also reported that many people with depression may go undiagnosed because of shame or a mistaken belief that they have another medical condition, so we do not observe them (16). Thirdly, sleep duration was self-reported by participants, and nap duration was converted by a categorical variable, which may lead to recall bias. Consequently, the total sleep duration in our study might be overestimated or underestimated. An objective sleep measure such as ActiGraph would have been preferable (43), but it is often needs to be more practical and financially prohibitive in extensive studies. Fourth, we did not use a standard scale to measure sleep problems, such as Jenkins Sleep Problems Scale (44). However, the method used to classify sleep problems in this study has been used in previous studies and has worked well (18). Sleeping pills or drugs using were recorded to assess sleep quality (45) and then explored the relationship with depression. However, few studies have explored the relationship between the use of sleeping pills or drugs and depression, so we could not make comparisons. Finally, although many covariates were adjusted in our study, there were other existing confounders, for example, the degree of physical fitness and diet. Despite the limitations, the study provides valuable information on the association between sleep and depression in the northwestern China. Furthermore, this study used RCS to identify the nonlinear relationship between sleep duration and depression. At last, the combined effect of sleep duration and sleep problems for depression was explored.

## 5. Conclusion

In summary, our study found that short sleep duration (< 7 h) and sleep problems were associated with an elevated risk of depression. We further found a nonlinear relationship between sleep duration and the risk of depression. Finally, our analyses showed that individuals reporting sleep problems were more likely to report depression than those without sleep problems in all three sleep duration groups. These findings provide valuable insights into the relationship between sleep and depression, which can inform future research and interventions aimed to improve mental health.

## Data availability statement

The original contributions presented in the study are included in the article/Supplementary material, further inquiries can be directed to the corresponding author.

## Ethics statement

The studies involving human participants were reviewed and approved by Human Research Ethics Committee of Xi’an Jiaotong University. The patients/participants provided their written informed consent to participate in this study.

## Author contributions

BM, HY, SD, and JL designed the research. JL analyzed the data and wrote the paper. BM and JL had primary responsibility for the final content. SC, YH, HL, YW, BZ, KX, PY, HY, LZ, and SD provided additional interpretation of the data and revisions to the manuscript. All authors contributed to the article and approved the submitted version.

## Funding

This work was supported by the National Natural Science Foundation of China (Grant number: 82103944), National Key Research and Development Program (Grant numbers: 2017YFC0907200 and 2017YFC0907201), and The Science and Technology Resources Open Sharing Platform of the Shaanxi Province (Grant number: 2023-CX-PT-47). We are grateful to research grant funding from National Key R&D Program of China (Grant numbers: 2017YFC0907200, 2017YFC0907201, 2017YFC0907202, 2017YFC0907203, 2017YFC0907204, and 2017YFC0907205) since 2017.

## Conflict of interest

The authors declare that the research was conducted in the absence of any commercial or financial relationships that could be construed as a potential conflict of interest.

## Publisher’s note

All claims expressed in this article are solely those of the authors and do not necessarily represent those of their affiliated organizations, or those of the publisher, the editors and the reviewers. Any product that may be evaluated in this article, or claim that may be made by its manufacturer, is not guaranteed or endorsed by the publisher.
