# Supplementary material for: Association of sleep behavior with depression: a cross-sectional study in northwestern China
Source: Front Psychiatry. 2023 Jun 23;14:1171310. doi: 10.3389/fpsyt.2023.1171310 (PMC10327479; doi:10.3389/fpsyt.2023.1171310)
Supplement: Supplementary file 1 [file Table_1.DOCX]

Supplementary Material

Association of Sleep Behavior with Depresson: a cross-sectional study in Northwest China

# Jingchun LIU, Suixia CAO, Yating HUO, Huimeng LIU, Yutong WANG, Binyan ZHANG, Kun XU, Peiying YANG, Lingxia ZENG, Shaonong DANG, Hong YAN, Baibing MI^*^

*** Correspondence:** Baibing MI: [xjtu.mi@xjtu.edu.cn](mailto:xjtu.mi@xjtu.edu.cn)

# Supplementary Figures and Tables

## Supplementary Figures


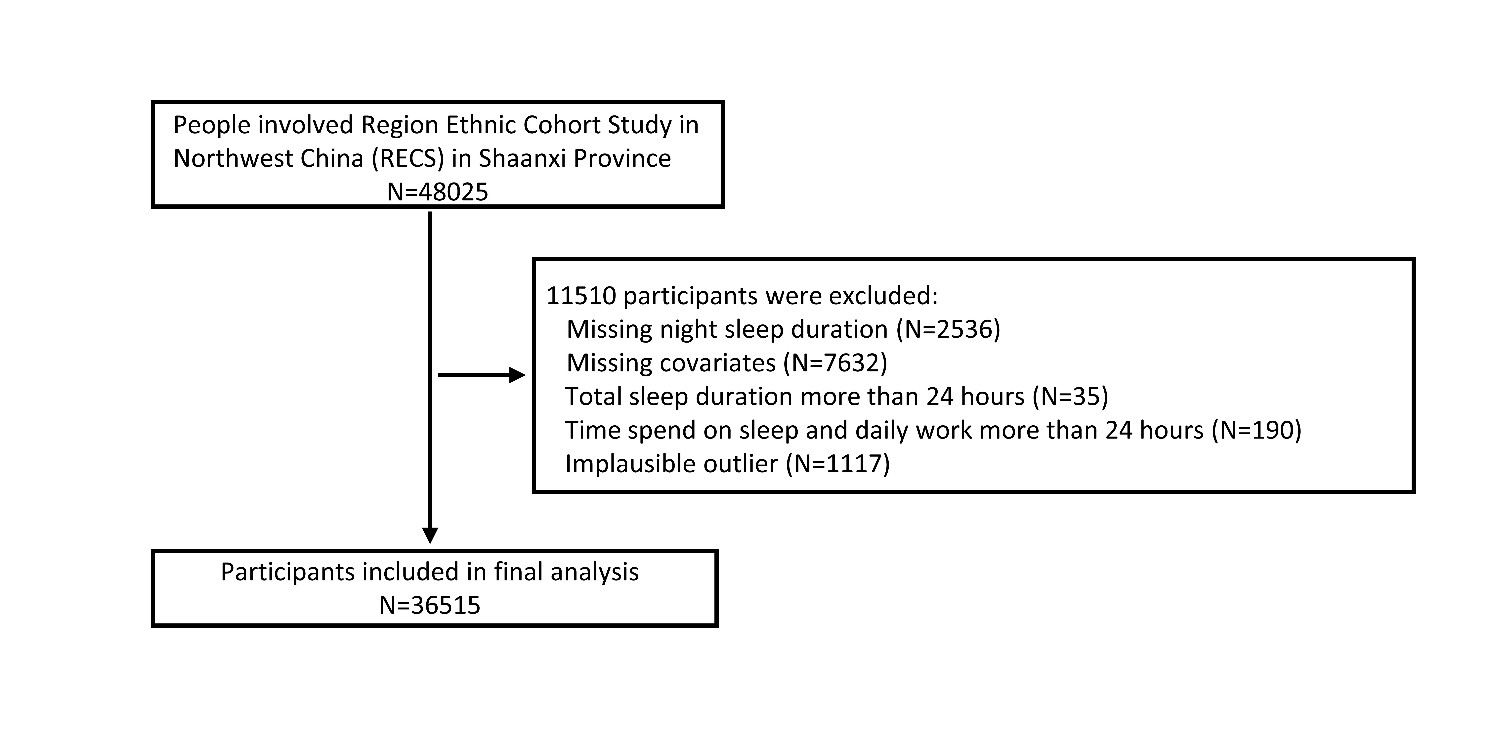


**Supplementary Figure 1.** Flow diagram of the study population.

## Supplementary Tables

**Table 1.** Questions and definitions of sleep problems

| Question | Sleep problems definitions |
| --- | --- |
| Having trouble falling asleep (sleep onset latency ≥ 30 min) after going to bed or waking up in the middle of the night at least 3 days a week | Difficulty initiating and maintaining sleep |
| Waking up too early and not be able to get back to sleep at least 3 days a week | Early morning awaking |
| Use of sleep pills or drugs to help sleep because of bad sleep at least 1 day a week | Use of sleeping pills or drugs |
| Having trouble keeping sober-minded during daytime because of bad sleep at least 3 days a week | Daytime dysfunction |
| One or more of the four afore mentioned sleep problems | Any sleep problems |

| **Table 2. The effect of sleep duration for depressive disorder by age group^a^** | | | | |
| --- | --- | --- | --- | --- |
|  | **≤60** | | **>60** | |
|  | **OR (95%CI)** | **P value** | **OR (95%CI)** | **P value** |
| sleep duration |  |  |  |  |
| 7~9 | Reference | | Reference | |
| <7 | 2.11(1.20,3.73) | **0.010** | 2.01(0.68,5.91) | 0.205 |
| >9 | 1.14(0.66,1.96) | 0.648 | 1.79(0.63,5.08) | 0.275 |
| difficulty initiating and maintaining sleep |  |  |  |  |
| no | Reference | | Reference | |
| yes | 3.25(2.42,4.37) | **<.001** | 2.44(1.32,4.50) | **0.004** |
| early morning waking |  |  |  |  |
| no | Reference | | Reference | |
| yes | 2.80(2.03,3.85) | **<.001** | 2.51(1.32,4.75) | **0.005** |
| use of sleeping pills or drugs |  |  |  | |
| no | Reference | | Reference | |
| yes | 9.03(5.71,14.27) | **<.001** | 6.01(2.83,12.75) | **<.001** |
| daytime dysfunction |  |  |  |  |
| no | Reference | | Reference | |
| yes | 3.61(2.59,5.04) | **<.001** | 4.13(1.92,8.9) | **<.001** |
| Any sleep disturbances |  |  |  |  |
| no | Reference | | Reference | |
| yes | 4.27(3.12,5.85) | **<.001** | 2.81(1.52,5.21) | **0.001** |
| ^a^Analyses were conducted in the full model. | | | | |

| **Table 3. The effect of sleep duration for depressive disorder by location group^a^** | | | | |
| --- | --- | --- | --- | --- |
|  | **Rural** | | **Urban** | |
|  | **OR (95%CI)** | **P value** | **OR (95%CI)** | **P value** |
| sleep duration |  |  |  |  |
| 7~9 | Reference | | Reference | |
| <7 | 4.54(1.35,15.19) | **0.014** | 1.81(1.03,3.18) | **0.039** |
| >9 | 1.94(0.58,6.49) | 0.280 | 1.17(0.68,1.99) | 0.571 |
| difficulty initiating and maintaining sleep | | |  |  |
| no | Reference | | Reference | |
| yes | 2.22(1.22,4.05) | **0.009** | 3.37(2.50,4.55) | **<.001** |
| early morning waking | |  |  |  |
| no | Reference | | Reference | |
| yes | 2.27(1.18,4.34) | **0.014** | 2.88(2.09,3.96) | **<.001** |
| use of sleeping pills or drugs | |  |  | |
| no | Reference | | Reference | |
| yes | 10.37(4.99,21.55) | **<.001** | 7.41(4.64,11.84) | **<.001** |
| daytime dysfunction | |  |  |  |
| no | Reference | | Reference | |
| yes | 5.46(2.77,10.77) | **<.001** | 3.44(2.45,4.82) | **<.001** |
| Any sleep disturbances | |  |  |  |
| no | Reference | | Reference | |
| yes | 3.76(2.12,6.68) | **<.001** | 4.04(2.93,5.56) | **<.001** |
| ^a^Analyses were conducted in the full model. | | | | |

| Table 4. The effect of sleep duration for depressive disorder by season group of interview^a^ | | | | | | | | |
| --- | --- | --- | --- | --- | --- | --- | --- | --- |
|  | **spring** | | **summer** | | **autumn** | | **winter** | |
|  | **OR (95%CI)** | **P value** | **OR (95%CI)** | **P value** | **OR (95%CI)** | **P value** | **OR (95%CI)** | **P value** |
| sleep duration |  |  |  |  |  |  |  |  |
| 7~9 | Reference | | Reference | | Reference | | Reference | |
| <7 | 2.10(0.59,7.46) | 0.253 | 2.64(1.24,5.63) | **0.012** | 1.59(0.68,3.73) | 0.283 | 1.53(0.43,5.51) | 0.513 |
| >9 | 1.19(0.36,3.91) | 0.769 | 1.43(0.68,3) | 0.343 | 1.11(0.5,2.47) | 0.793 | 0.82(0.25,2.68) | 0.747 |
| difficulty initiating and maintaining sleep | | |  |  |  |  |  |  |
| no | Reference | | Reference | | Reference | | Reference | |
| yes | 2.89(1.4,5.94) | **0.004** | 3.82(2.6,5.61) | **<.001** | 2.71(1.71,4.31) | **<.001** | 1.68(0.73,3.86) | 0.221 |
| early morning waking | |  |  |  |  |  |  |  |
| no | Reference | | Reference | | Reference | | Reference | |
| yes | 3.52(1.66,7.44) | **0.001** | 2.28(1.49,3.5) | **<.001** | 3.45(2.14,5.56) | **<.001** | 2.24(0.93,5.39) | **0.071** |
| use of sleeping pills or drugs | |  |  | |  |  |  |  |
| no | Reference | | Reference | | Reference | | Reference | |
| yes | 9.52(3.11,29.14) | **<.001** | 7.65(4.26,13.71) | **<.001** | 7.72(3.82,15.62) | **<.001** | 10.75(3.65,31.64) | **<.001** |
| daytime dysfunction | |  |  |  |  |  |  |  |
| no | Reference | | Reference | | Reference | | Reference | |
| yes | 3.06(1.26,7.44) | **0.013** | 3.24(2.04,5.15) | **<.001** | 5.11(3.12,8.35) | **<.001** | 2.83(1.08,7.41) | **0.034** |
| Any sleep disturbances | |  |  |  |  |  |  |  |
| no | Reference | | Reference | | Reference | | Reference | |
| yes | 2.88(1.4,5.95) | **0.004** | 4.62(3.07,6.94) | **<.001** | 3.45(2.15,5.55) | **<.001** | 4.32(1.83,10.22) | **<.001** |
| ^a^Analyses were conducted in the full model. | | | | | | | | |

**Table 5.** Odds ratios (95% CI) of depression across sleep duration and sleep problem for participants reporting sleep duration between 4h and 12h

|  | **Model 1** | | **Model2** | | **Model 3** | |
| --- | --- | --- | --- | --- | --- | --- |
|  | **OR (95%CI)** | **P value** | **OR (95%CI)** | **P value** | **OR (95%CI)** | **P value** |
| **Sleep duration** | |  |  |  |  |  |
| **7~9** | Reference | |  | | Reference | |
| **<7** | 1.40 (1.03,1.89) | 0.032 | 1.63 (1.2,2.22) | 0.002 | 1.61 (1.19,2.18) | 0.002 |
| **≥9** | 0.50 (0.29,0.84) | 0.009 | 0.73 (0.43,1.24) | 0.246 | 0.72 (0.43,1.22) | 0.226 |
| **Difficulty initiating and maintaining sleep** | | | |  |  |  |
| no | Reference | |  | | Reference | |
| yes | 3.86 (2.92,5.09) | <.001 | 3.39 (2.57,4.47) | <.001 | 3.36 (2.54,4.43) | <.001 |
| **Early morning awaking** | |  |  |  |  |  |
| no | Reference | |  | | Reference | |
| yes | 3.25 (2.41,4.37) | <.001 | 2.95 (2.19,3.97) | <.001 | 2.96 (2.2,3.97) | <.001 |
| **Use of sleeping pills or drugs** | | |  |  |  |  |
| no | Reference | |  | | Reference | |
| yes | 7.70 (5.18,11.46) | <.001 | 8.76 (5.82,13.2) | <.001 | 8.84 (5.87,13.31) | <.001 |
| **Daytime dysfunction** | |  |  |  |  |  |
| no | Reference | |  | | Reference | |
| yes | 5.43 (3.98,7.41) | <.001 | 3.99 (2.91,5.47) | <.001 | 3.94 (2.87,5.39) | 0.001 |
| **Any sleep problems** | |  |  |  |  |  |
| no | Reference | |  | | Reference | |
| yes | 5.28 (3.94,7.07) | <.001 | 4.30 (3.21,5.76) | <.001 | 4.28 (3.2,5.74) | 0.001 |
| OR=odds ratio; CI=confidence interval  Model 1: crude model. Model 2: adjusted for sex, age, BMI, career, household income, and educational level.  Model 3: additionally adjusted drinking, smoking, tea, coffee, nightshift, and physical activity | | | | | | |

**Table 6.** Parameter estimate of sleep duration after multiple imputation^a^

| Sleep duration | Estimate | Std Error | 95% Confidence Limits | | DF | Minimum | Maximum | Theta0 | t for H0: | Pr > \|t\| |
| --- | --- | --- | --- | --- | --- | --- | --- | --- | --- | --- |
|  |  |  |  |  |  |  |  |  | Parameter=Theta0 |  |
| 7~9 |  |  |  |  |  |  |  |  |  |  |
| <7 | 0.42 | 0.10 | 0.22 | 0.61 | 2800000000.00 | 0.41 | 0.42 | 0 | 4.17 | <.001 |
| ≥9 | -0.35 | 0.14 | -0.63 | -0.08 | 924000000.00 | -0.36 | -0.35 | 0 | -2.56 | 0.010 |

^a^Analyses were conducted in the full model.

| **Table 7.** Odds ratios (95% CI) of depression across sleep duration and sleep problem for all participants | | | | | | |
| --- | --- | --- | --- | --- | --- | --- |
|  | **Model 1** | | **Model2** | | **Model 3** | |
|  | **OR (95%CI)** | **P value** | **OR (95%CI)** | **P value** | **OR (95%CI)** | **P value** |
| **Sleep duration** | |  |  |  |  |  |
| **7~9** | Reference | | Reference | | Reference | |
| **<7** | 1.44 (1.07,1.93) | 0.015 | 1.71 (1.26,2.31) | 0.001 | 1.69 (1.25,2.28) | 0.001 |
| **≥9** | 0.54 (0.32,0.89) | 0.017 | 0.79 (0.47,1.32) | 0.371 | 0.78 (0.47,1.31) | 0.354 |
| **Difficulty initiating and maintaining sleep** | | | |  |  |  |
| no | Reference | | Reference | | Reference | |
| yes | 3.53 (2.69,4.64) | <.001 | 3.15 (2.39,4.15) | <.001 | 3.13 (2.37,4.13) | <.001 |
| **Early morning awaking** | |  |  |  |  |  |
| no | Reference | | Reference | | Reference | |
| yes | 2.95 (2.2,3.96) | <.001 | 2.75 (2.04,3.69) | <.001 | 2.75 (2.05,3.71) | <.001 |
| **Use of sleeping pills or drugs** | | |  |  |  |  |
| no | Reference | | Reference | | Reference | |
| yes | 6.96 (4.7,10.3) | <.001 | 8.1 (5.37,12.19) | <.001 | 8.15 (5.4,12.29) | <.001 |
| **Daytime dysfunction** | |  |  |  |  |  |
| no | Reference | | Reference | | Reference | |
| yes | 4.97 (3.65,6.75) | <.001 | 3.74 (2.73,5.13) | <.001 | 3.70 (2.69,5.07) | 0.001 |
| **Any sleep problems** | |  |  |  |  |  |
| no | Reference | | Reference | | Reference | |
| yes | 4.90 (3.68,6.51) | <.001 | 4.05 (3.03,5.41) | <.001 | 4.04 (3.02,5.4) | 0.001 |
| OR=odds ratio; CI=confidence interval  Model 1: crude model. Model 2: adjusted for sex, age, BMI, career, household income, and educational level. Model 3: additionally adjusted drinking, smoking, tea, coffee, nightshift, and physical activity. | | | | | | |

**Table 8.** The combination effect of sleep duration and sleep problems for depression

| **combination of sleep duration and sleep problems** | **Model 1** | | **Model2** | | **Model 3** | |
| --- | --- | --- | --- | --- | --- | --- |
|  | **OR (95%CI)** | **P value** | **OR (95%CI)** | **P value** | **OR (95%CI)** | **P value** |
| **7~9** |  |  |  |  |  |  |
| without sleep problems | Reference | | Reference | | Reference | |
| with sleep problems | 4.63 (3.23,6.62) | <.001 | 3.50 (2.42,5.05) | <.001 | 3.44 (2.38,4.98) | <.001 |
| **<7** |  |  |  |  |  |  |
| without sleep problems | Reference | | Reference | | Reference | |
| with sleep problems | 4.40 (2.36,8.21) | <.001 | 4.46 (2.39,8.35) | <.001 | 4.61 (2.46,8.65) | <.001 |
| **≥9** |  |  |  |  |  |  |
| without sleep problems | Reference | | Reference | | Reference | |
| with sleep problems | 9.31 (3.53,24.54) | <.001 | 5.88 (2.1,16.41) | <.001 | 5.65 (1.97,16.19) | 0.001 |
| OR=odds ratio; CI=confidence interval  Model 1: crude model. Model 2: adjusted for sex, age, BMI, career, household income, and educational level. Model 3: additionally adjusted drinking, smoking, tea, coffee, nightshift, and physical activity. | | | | | | |

| **Table 9. The effect of sleep duration for depressive disorder by age group^a^** | | | | |
| --- | --- | --- | --- | --- |
|  | **≤60** | | **>60** | |
|  | **OR (95%CI)** | **P value** | **OR (95%CI)** | **P value** |
| sleep duration |  |  |  |  |
| 7~9 | Reference | | Reference | |
| <7 | 2.17(1.2,3.92) | **0.010** | 2.28(0.64,8.12) | 0.202 |
| >9 | 1.17(0.66,2.06) | 0.587 | 2.06(0.6,7.05) | 0.249 |
| difficulty initiating and maintaining sleep |  |  |  |  |
| no | Reference | | Reference | |
| yes | 3.25(2.4,4.41) | **<.001** | 2.45(1.24,4.84) | **0.010** |
| early morning waking |  |  |  |  |
| no | Reference | | Reference | |
| yes | 2.78(2.01,3.86) | **<.001** | 2.48(1.22,5.05) | **0.012** |
| use of sleeping pills or drugs |  |  |  | |
| no | Reference | | Reference | |
| yes | 8.94(5.59,14.29) | **<.001** | 5.94(2.58,13.65) | **<.001** |
| daytime dysfunction |  |  |  |  |
| no | Reference | | Reference | |
| yes | 3.6(2.56,5.06) | **<.001** | 4.01(1.7,9.44) | **0.002** |
| Any sleep disturbances |  |  |  |  |
| no | Reference | | Reference | |
| yes | 4.29(3.11,5.93) | **<.001** | 2.86(1.44,5.68) | **0.003** |
| ^a^Analyses were conducted in the full model. | | | | |

| **Table 10. The effect of sleep duration for depressive disorder by location group^a^** | | | | |
| --- | --- | --- | --- | --- |
|  | **Rural** | | **Urban** | |
|  | **OR (95%CI)** | **P value** | **OR (95%CI)** | **P value** |
| sleep duration |  |  |  |  |
| 7~9 | Reference | | Reference | |
| <7 | 5.57(1.29,24.08) | **0.021** | 1.86(1.03,3.34) | **0.039** |
| >9 | 2.38(0.55,10.26) | 0.244 | 1.2(0.69,2.09) | 0.520 |
| difficulty initiating and maintaining sleep |  |  |  |  |
| no | Reference | | Reference | |
| yes | 2.19(1.13,4.26) | **0.021** | 3.44(2.53,4.68) | **<.001** |
| early morning waking |  |  |  |  |
| no | Reference | | Reference | |
| yes | 2.2(1.06,4.56) | **0.034** | 2.87(2.07,3.99) | **<.001** |
| use of sleeping pills or drugs |  |  |  | |
| no | Reference | | Reference | |
| yes | 10.1(4.5,22.67) | **<.001** | 7.35(4.54,11.9) | **<.001** |
| daytime dysfunction |  |  |  |  |
| no | Reference | | Reference | |
| yes | 5.31(2.48,11.36) | **<.001** | 3.43(2.42,4.85) | **<.001** |
| Any sleep disturbances |  |  |  |  |
| no | Reference | | Reference | |
| yes | 3.79(2,7.17) | **<.001** | 4.06(2.92,5.64) | **<.001** |
| ^a^Analyses were conducted in the full model. | | | | |

| **Table 11. The effect of sleep duration for depressive disorder by interview quarter group^a^** | | | | | | | | |
| --- | --- | --- | --- | --- | --- | --- | --- | --- |
|  | **spring** | | **summer** | | **autumn** | | **winter** | |
|  | **OR (95%CI)** | **P value** | **OR (95%CI)** | **P value** | **OR (95%CI)** | **P value** | **OR (95%CI)** | **P value** |
| sleep duration |  |  |  |  |  |  |  |  |
| 7~9 | Reference | | Reference | | Reference | | Reference | |
| <7 | 2.48(0.5,12.33) | 0.266 | 2.80(1.24,6.32) | 0.013 | 1.69(0.66,4.29) | 0.273 | 1.80(0.33,9.79) | 0.499 |
| >9 | 1.43(0.32,6.48) | 0.639 | 1.52(0.69,3.37) | 0.300 | 1.19(0.49,2.86) | 0.699 | 0.97(0.2,4.68) | 0.971 |
| difficulty initiating and maintaining sleep | | |  |  |  |  |  |  |
| no | Reference | | Reference | | Reference | | Reference | |
| yes | 2.92(1.26,6.78) | **0.012** | 3.83(2.56,5.73) | **<.001** | 2.72(1.67,4.45) | **<.001** | 1.65(0.59,4.63) | 0.339 |
| early morning waking | |  |  |  |  |  |  |  |
| no | Reference | | Reference | | Reference | | Reference | |
| yes | 3.60(1.5,8.63) | **0.004** | 2.26(1.44,3.54) | **<.001** | 3.45(2.08,5.73) | **<.001** | 2.20(0.74,6.48) | 0.155 |
| use of sleeping pills or drugs | |  |  | |  |  |  |  |
| no | Reference | | Reference | | Reference | | Reference | |
| yes | 10.35(2.75,38.92) | **0.001** | 7.50(4.06,13.84) | **<.001** | 7.63(3.6,16.15) | **<.001** | 11.85(3.27,42.9) | **<.001** |
| daytime dysfunction | |  |  |  |  |  |  |  |
| no | Reference | | Reference | | Reference | | Reference | |
| yes | 3.01(1.06,8.52) | **0.038** | 3.21(1.98,5.2) | **<.001** | 5.14(3.05,8.65) | **<.001** | 2.73(0.84,8.89) | 0.096 |
| Any sleep disturbances | |  |  |  |  |  |  |  |
| no | Reference | | Reference | | Reference | | Reference | |
| yes | 2.97(1.27,6.92) | 0.012 | 4.66(3.04,7.14) | **<.001** | 3.50(2.11,5.81) | **<.001** | 4.75(1.61,13.98) | **<.001** |
| ^a^Analyses were conducted in the full model. | | | | | | | | |

**Table 12.** Odds ratios (95% CI) of depression across sleep duration and sleep problem for participants reporting sleep duration between 4h and 12h

|  | **Model 1** | | **Model2** | | **Model 3** | |
| --- | --- | --- | --- | --- | --- | --- |
|  | **OR (95%CI)** | **P value** | **OR (95%CI)** | **P value** | **OR (95%CI)** | **P value** |
| **Sleep duration** | |  |  |  |  |  |
| **7~9** | Reference | | Reference | | Reference | |
| **<7** | 1.39 (1.02,1.89) | 0.035 | 1.63 (1.19,2.22) | 0.002 | 1.60 (1.17,2.19) | 0.003 |
| **≥9** | 0.48 (0.28,0.82) | 0.008 | 0.71 (0.41,1.22) | 0.217 | 0.70 (0.41,1.21) | 0.202 |
| **Difficulty initiating and maintaining sleep** | | | |  |  |  |
| no | Reference | | Reference | | Reference | |
| yes | 3.85 (2.92,5.09) | <.001 | 3.39 (2.56,4.49) | <.001 | 3.36 (2.53,4.46) | <.001 |
| **Early morning waking** | |  |  |  |  |  |
| no | Reference | | Reference | | Reference | |
| yes | 3.24 (2.4,4.36) | <.001 | 2.94 (2.18,3.98) | <.001 | 2.95 (2.18,3.99) | <.001 |
| **Use of sleeping pills or drugs** | | |  |  |  |  |
| no | Reference | | Reference | | Reference | |
| yes | 7.6 (5.09,11.34) | <.001 | 8.68 (5.72,13.18) | <.001 | 8.77 (5.77,13.34) | <.001 |
| **Daytime dysfunction** | |  |  |  |  |  |
| no | Reference | | Reference | | Reference | |
| yes | 5.41 (3.96,7.38) | <.001 | 3.98 (2.89,5.48) | <.001 | 3.92 (2.84,5.41) | 0.001 |
| **Any sleep problems** | |  |  |  |  |  |
| no | Reference | | Reference | | Reference | |
| yes | 5.3 (3.95,7.1) | <.001 | 4.32 (3.2,5.81) | <.001 | 4.3 (3.19,5.8) | 0.001 |
| OR=odds ratio; CI=confidence interval  Model 1: crude model. Model 2: adjusted for sex, age, BMI, career, household income, and educational level.  Model 3: additionally adjusted drinking, smoking, tea, coffee, nightshift, and physical activity | | | | | | |

**Table 13.** Parameter estimate of sleep duration after multiple imputation^a^

| Sleep duration | Estimate | Std Error | 95% Confidence Limits | | DF | Minimum | Maximum | Theta0 | t for H0:  Parameter=Theta0 | Pr > \|t\| |
| --- | --- | --- | --- | --- | --- | --- | --- | --- | --- | --- |
| 7~9 |  |  |  |  |  |  |  |  |  |  |
| <7 | 0.42 | 0.10 | 0.22 | 0.62 | 3120000000.00 | 0.42 | 0.42 | 0 | 4.14 | <.001 |
| ≥9 | -0.37 | 0.14 | -0.64 | -0.09 | 1030000000.00 | -0.37 | -0.36 | 0 | -2.58 | 0.010 |

^a^ All analyses were conducted in the full model.
